# Reasons for extraction in primary teeth among 5-12 years school children in Haryana, India- A cross-sectional study

**DOI:** 10.4317/jced.53076

**Published:** 2017-04-01

**Authors:** Mohit Bansal, Nidhi Gupta, Preety Gupta, Vikram Arora, Sahil Thakar

**Affiliations:** 1 MDS, Assistant Professor, Department of Public Health Dentistry Swami Devi Dyal Hospital and Dental College Barwala, Haryana; 2 MDS, Professor, Department of Public Health Dentistry Swami Devi Dyal Hospital and Dental College Barwala, Haryana; 3 MDS, Lecturer, Department of Public Health Dentistry Swami Devi Dyal Hospital and Dental College Barwala, Haryana

## Abstract

**Background:**

Due to high prevalence of oral diseases extraction of primary teeth is a common and a major concern in developing countries. These teeth are given least importance as they are believed to shed off automatically, thus leading to serious problems like crowding and malocclusion.

**Material and Methods:**

A cross sectional study was carried out among children aged 5 to 12 years among 1347 children. The data was recorded on a prestructured questionnaire. Reasons for extraction of teeth were based on Kay and Blinkhorn criteria.

**Results:**

20.4% children were having tooth loss due to various reasons. The main reason for extraction was found to be caries in 64.3% followed by trauma in maxillary teeth among 43.02% of children.

**Conclusions:**

Presence of early loss of primary teeth result in occlusal disturbances and space loss among children. Hence, proper treatment regimens must be followed by the dental professionals and should be the need of the hour.

** Key words:**Extraction, children, primary teeth, caries.

## Introduction

Human beings have two successive sets of teeth primary and permanent, therefore they are better known as diphodonts. Primary teeth previously were also known as deciduous or milk teeth which account for a total of 20 teeth (10 in each arch). These teeth begin to develop at 6 weeks of intrauterine life. The first primary tooth erupts in the oral cavity at an age of 6 months and this eruption sequence completes till the age of 2 years. These teeth are functional at the age of 5 years till 12 years after which the permanent teeth starts to erupt in the oral cavity (1).

Primary teeth are considered to be equally important as the permanent teeth. Primary teeth help in chewing of food, speech, and aesthetics and also act as a template for permanent teeth to assume proper position in the dental arch (2).

The early or premature loss is defined by the loss of primary tooth before the time of its natural exfoliation (3). It is believed that premature loss of primary teeth is related to space reduction and hence will result in malocclusion of successive teeth. The most common reason of premature loss of primary teeth is most commonly associated with dental caries (4,5). Although the prevalence of dental caries in young children has decreased considerably in recent years, caries continues to affect many children in the general population. Other causes may include trauma, ectopic eruption, congenital disorders, and arch length deficiencies causing resorption of primary teeth (6). The loss of primary teeth also predisposes crowding, rotation and impaction in the permanent teeth (7).

The access to oral health care is very limited especially in rural children. It is also believed that the primary teeth need no treatment as new teeth will erupt automatically and may be attributed to the fact that the parents lack in education and attitude towards dental treatment in primary teeth (8).

In order to facilitate planning for dental health services and to develop strategies to continue the reduction in tooth loss it is important to identify the factors which caused such loss. Despite the abundance of studies documenting reasons for extraction of permanent teeth in the literature, very little information is available describing the reasons for the extraction of primary teeth in the state of Haryana. In order to develop strategies to reduce primary tooth loss, it is important to understand the factors which lead to such loss and the relative contributing factors as reasons may vary geographically.

Hence, the main aim of this study was to investigate the various reasons for extraction in children with primary teeth aged 5-12 years in Barwala, Panchkula District, Haryana.

## Material and Methods

A cross sectional prospective study was carried out among children aged 5 to 12 years of age as a part of school dental health program. Of the total 28 government schools in Barwala block 1347 children were randomly selected in the study. The criteria for age were fulfilled from the school records. The children who had completed their 5th birthday were included in the age group of 5 years and similarly the rest of the age groups were categorized.

Sample size was estimated based on the pilot study done on 100 school children under the same criteria as mentioned. The main reason for tooth loss was found to be dental caries among 55% and using the same prevalence with 5% error and 95% confidence interval the sample size was determined which was found to be approximately 1250. To incorporate for the loss of ubjects the sample size was determined to be 1347.

The study was carried out for a period of ten months from July 2015 to February 2016. The study included three examiners (MB, VA, ST) who were calibrated prior to commencement of the study. The inter examiner reliability was checked by the standard examiner (MB) which was found out to be 0.79. Informed consent was taken from the parents prior to the start of the study. All the teeth present in the oral cavity were examined. The Questionnaire included demographic details of the children, socioeconomic status, detailed history, clinical examination and reasons for missing teeth were recorded on a pre-structured questionnaire. The questionnaire was pre-tested on 20 subjects who were not included in the final analysis. Based on the responses provided, a few minor modifications were subsequently made in the questionnaire, and its Cronbach alpha (α) was found out to be 0.87.

The exclusion criteria included children suffering from congenitally missing teeth and children with birth defects. Reasons for extraction of teeth were divided into the following categories based on those described by Kay and Blinkhorn (9).

1. Caries: Primary and secondary caries plus all sequelae including periapical abscess and failed pulpotomy.

2. Orthodontic: Tooth removed to prevent or correct malocclusion.

3. Trauma: Tooth extracted as a direct result of acute trauma.

4. Loss: Tooth extracted because of its mobility; time for exfoliation.

5. Periodontal disease: Loss of function, periodontal abscess and pain.

6. General medical reasons: Prophylactic extraction.

7. Economic reasons: The tooth could have been saved but the patient found treatment too expensive.

8. Over-retention: Prolonged retention of primary teeth.

9. Patient/parent request: The tooth could have been repaired, but the patient/parent insisted on extraction.

10. Other reasons: Teeth extracted for reasons not encompassed by any of the above categories.

The data was analyzed using SPSS version 20. The Chi square test and Student’s t-test (Unpaired) was used to find significant responses (*p*≤0.05), if any, whereas the Analysis of Variance (ANOVA) was used to find the significant difference between the means of the responses of the various variables assessed in the present study. In the end, Spearman’s correlation was used to find the correlation between the responses of the subjects and the demographic variables recorded.

## Results

A total of 1347 children were examined, aged 5 to 12 years (mean age 9.7±2.6 years), out of which 707 (52.5%) were males and 640 (47.5%) were females. It was found that 276 (20.4%) children were having tooth loss, out of which a total of 483 teeth were extracted due to many reasons which included dental caries, trauma, mobility, over retention and other factors like economic reasons and parent request. Demographic details of the subjects including age and gender wise distribution and socioeconomic status using BG Prasad classification is described in  Table 1.

**Table 1:**
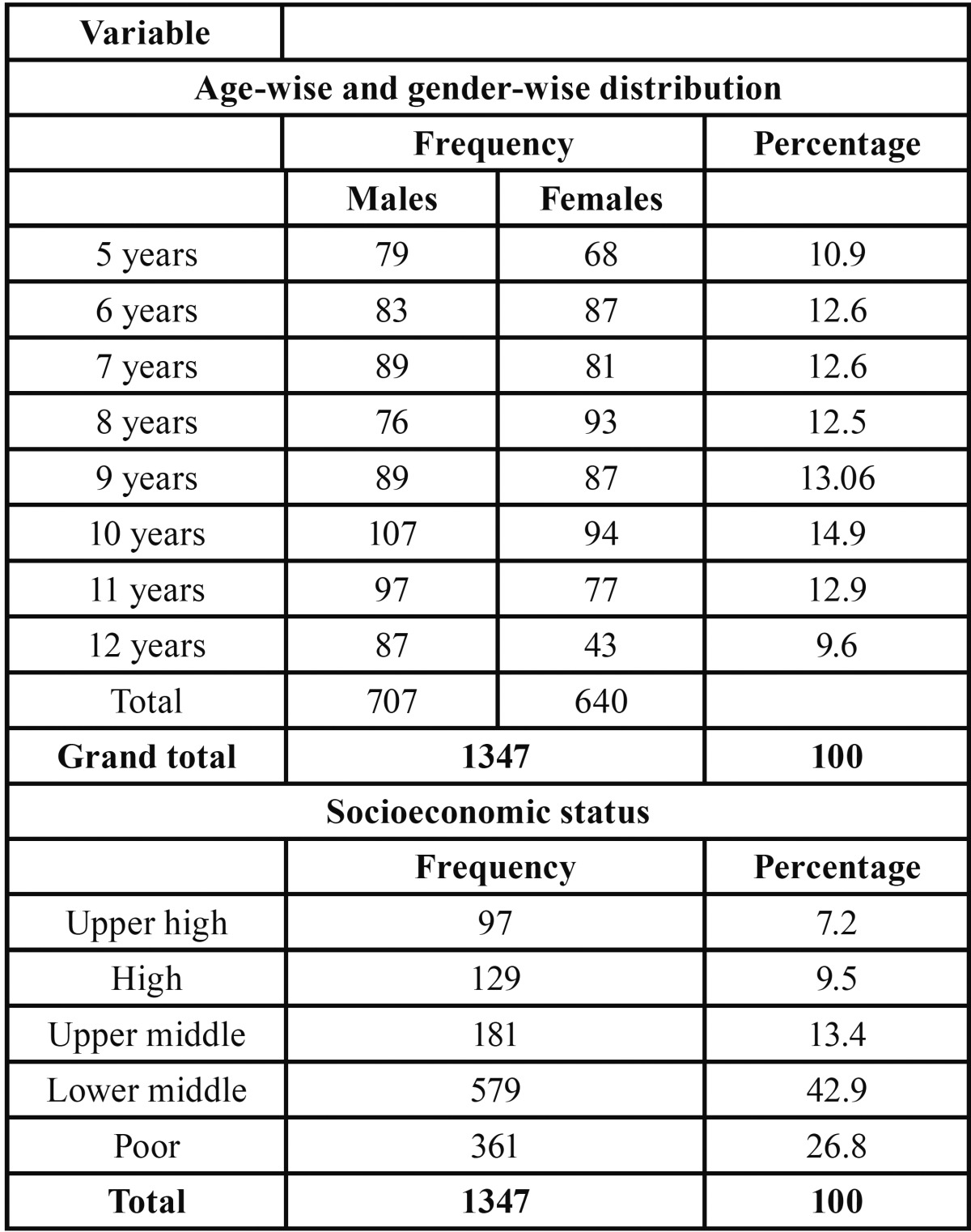
Showing demographic details of the subjects.

The most common reason for extraction among total of 483 teeth extracted was found to be dental caries in 311 teeth (64.3%) followed by trauma in 86 (17.8%) and mobility in 21 (4.3%) teeth. Over retention and orthodontic reasons accounted for 42 (8.6%) and 4(0.82%)loss of teeth, respectively. Since very few extractions were attributed to other category which included pain and abscess, economic reason, parent request accounted for only 19 (3.9%). Upon gender wise comparison, Chi square analysis revealed that a significant difference was seen between males and females with respect to dental caries ( Table 2).

**Table 2:**
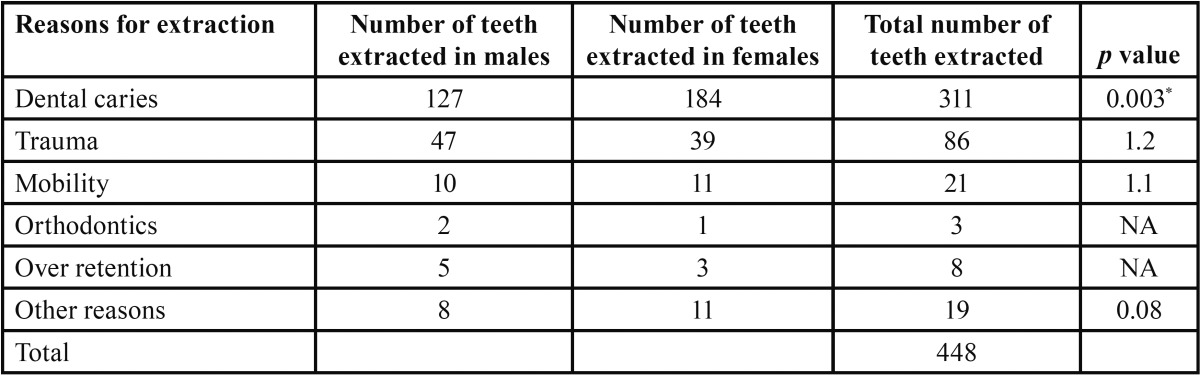
Showing reasons for extraction of teeth among children.

It was also found that the subjects belonging to lower middle and poor socioeconomic strata could not bear the treatment cost and above all believed that extraction is the better option for any decayed tooth as it is primary teeth require no treatment as permanent teeth are yet to come. In our study it was also found that parents often took these extracted teeth along with them as they offer these teeth to god and had a strong thought that the successor teeth will erupt healthy ( Table 3).

**Table 3:**
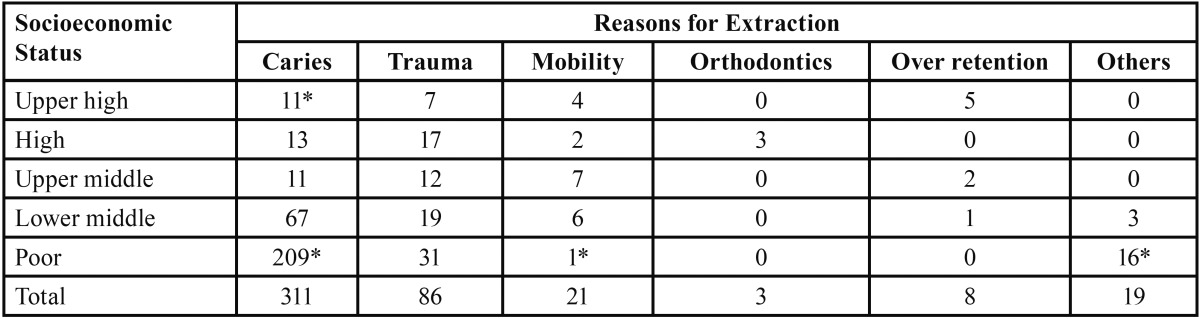
Showing comparison between socioeconomic status and reasons for extraction.

As age of the children progresses, it was found that the number of extractions also increased. Teeth extractions due to caries were maximum in the age of 12 years (24.7%) and extraction due to over retention was maximum in the age of 11 years (26.1%). Trauma was found to be the most common reason for extraction in 24.4% of children in the age group of 10 years ( Table 4). In agreement to our aforementioned observations, Spearman’s Correlation revealed that dental caries had a nearly perfect positive relationship (r=0.86) with increasing age of the children, as compared to other reasons which did not yield any significant correla-tion values. In both the arches maxillary and mandibular, the most common reason for extraction was found to be caries (58.1% and 77.2% respectively) followed by trauma (23.6% and 14.3% respectively). Out of a total of 276 children 131 (47.4%) had at least one tooth extracted. 83 (30.07%) children had more than one tooth extracted. the remaining 62 (22.4%) were having more than three teeth extracted. The main reason for extraction in 131 children was found to be caries while the reason behind extrac-tion of more than three teeth was trauma, mobility and caries. The gender distribution of the patients with one or more primary teeth extracted was 146 (53.2%) males and 130 (46.8%) females, illustrating that there was no statistical difference found (*p*≤0.339).

**Table 4:**
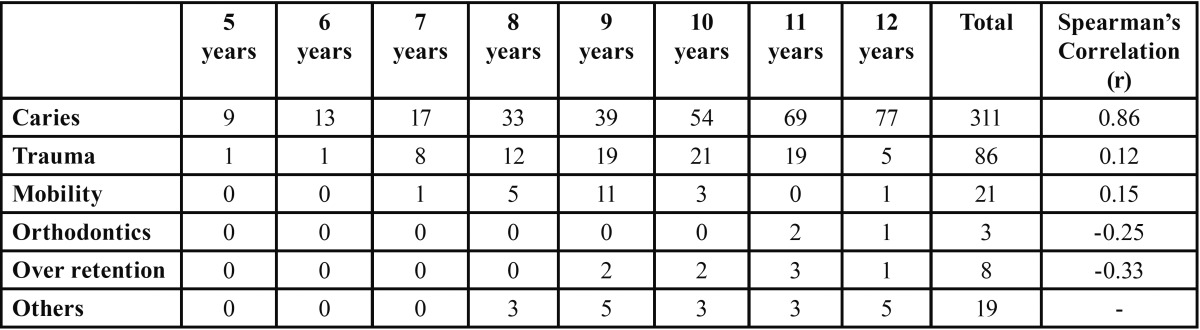
Showing distribution of extraction indicators among children according to age.

## Discussion

Preservation of primary teeth is one of the major concerns for dentists. These teeth serve as a jewel which increases the smile and hence the self-esteem of the children. A total of 1347 children aged 5-12 years in Barwala, District Panchkula were included in the present study, out of which 276 (20.4%) children had their tooth loss. The study was undertaken as a part of school dental health programs in different government schools of Barwala. This high percentage of primary teeth loss could be due to the lack of knowledge among parents or a belief that the primary teeth will ultimately be replaced again.

In the study the main reason of extraction among teeth was found to be caries (64.3%). It is a known fact that small children do not necessarily complain of pain, however they do manifest the effects of pain in their altered eating and inability to sleep properly (10).

In our study it was found that primary maxillary and mandibular first molars were more affected due to caries than second molars. This was in contrary with the study done by Alamoundi N (11). The reason might be that the primary first molar erupts first than second molar and hence is present in the oral cavity for a longer duration of time. Premature loss of a primary molar tooth due to caries also results in not only loss of function, but also can lead to increased possibility that the other teeth may drift (12). This may influences the occlusion normal development and creates an increased need for orthodontic treatment (13).

Extraction due to trauma also increased with age. In the present study it was found that the boys are more affected by trauma than girls, which corroborates the findings of other studies by Cavalcanti AL *et al.* (14). This may be attributed to the behavioral factors, with the children of this age tend to be more energetic and inclined toward vigorous outdoor activities. In our study, primary maxillary central incisor was found to be the most affected tooth due to trauma. It was similar to the studies done by done by Altun C *et al.* (15) and Ferreira JM *et al.* (16). It is well documented that accidental fall due while playing and overjet greater than 3 mm were two and a half times more at risk compared with individuals who had a normal overjet (14,15).

The study was conducted in government school children in Barwala where the main limitations were found to be low access to oral health care, lack of education and attitude of the children and parents towards oral health. Therefore, it is mandatory to provide oral health education among school children and parents. School teachers should also be educated for emergency oral care.

## Conclusions

Premature loss of teeth is a cause of great concern for the dentists and parents to reduce problems of malocclusion and space closure. Hence, the dental professionals must take necessary steps at an initial stage by treating the affected teeth and hence space for the successors to erupt be preserved.
